# Supplementary material for: Comparative genomics provides new insights into the diversity, physiology, and sexuality of the only industrially exploited tremellomycete: Phaffia rhodozyma
Source: BMC Genomics. 2016 Nov 9;17:901. doi: 10.1186/s12864-016-3244-7 (PMC5103461; doi:10.1186/s12864-016-3244-7)
Supplement: Additional file 6: — List of orphan genes with links to PFAM (related to Additional file 1: Table S1). (ZIP 1428 kb) [file 12864_2016_3244_MOESM6_ESM.zip › PFAM_UNIQUE/G00333_P.html]

#                                                                            --- full sequence --- -------------- this domain -------------   hmm coord   ali coord   env coord
  
# target name        accession   tlen query name           accession   qlen   E-value  score  bias   #  of  c-Evalue  i-Evalue  score  bias  from    to  from    to  from    to  acc description of target
  
#------------------- ---------- ----- -------------------- ---------- ----- --------- ------ ----- --- --- --------- --------- ------ ----- ----- ----- ----- ----- ----- ----- ---- ---------------------
  
G00333\_P             -            243 CENP-B\_dimeris       PF09026.5    101      0.14   11.1   8.2   1   2       3.4   5.9e+02   -0.5   0.0    24    38    29    43    12    65 0.61 - Centromere protein B dimerisation domain  
G00333\_P             -            243 CENP-B\_dimeris       PF09026.5    101      0.14   11.1   8.2   2   2    0.0013      0.23   10.4   6.6    10    46   128   164   120   171 0.67 - Centromere protein B dimerisation domain
